# Citicoline: A Food Beneficial for Patients Suffering from or Threated with Glaucoma

**DOI:** 10.3389/fnagi.2016.00073

**Published:** 2016-04-08

**Authors:** Pawel Grieb, Anselm Jünemann, Marek Rekas, Robert Rejdak

**Affiliations:** ^1^Department of Experimental Pharmacology, Mossakowski Medical Research Centre, Polish Academy of SciencesWarsaw, Poland; ^2^Department of Ophthalmology, University of RostockRostock, Germany; ^3^Department of Ophthalmology, Military Institute of MedicineWarsaw, Poland; ^4^Department of General Ophthalmology, Medical University of LublinLublin, Poland

**Keywords:** citicoline, primary open angle glaucoma, neuroprotection

## Abstract

Oral form of citicoline, a nootropic and neuroprotective drug in use for almost five decades, recently was pronounced a food supplement in both USA and EU. The idea of adding citicoline to topical treatment of primary open angle glaucoma (POAG) aimed at decreasing intraocular pressure (IOP) appeared as a logical consequence of accepting neurodegenerative character of this disease. Experimental data, and also few clinical studies indicate that this substance has potential to counteract some important pathological mechanisms which seem to contribute to POAG initiation and progression, such as excitotoxicity and oxidative stress.

## Introduction

Citicoline is the INN (International Non-proprietary Name) of cytidine-diphosphocholine (CDP-Choline) marketed in several countries as an injectable nootropic drug since 1970’s. Because of lack of toxicity and perfect tolerance recently oral citicoline has been authorized as a food ingredient in both USA and the European Union (for details see Grieb, 2015a). In 2002 two of us reviewed citicoline pharmacodynamics and pointed to its beneficial effects in glaucoma, which at that time were thought to be related mainly to stimulation of intracellular synthesis of phosphatidycholine (Grieb and Rejdak, 2002). Since then many new important findings have been published concerning both glaucoma and citicoline, which prompted us to review this subject once more.

## Glaucoma: Clinical Landscape

The term “glaucoma” or “glaucomatous optic neuropathy” (GON) describes a chronic human ophthalmic disease, in the course of which the neuroretinal rim of the optic nerve becomes progressively thinner and the optic nerve cup enlarges (Kwon et al., 2009). Worldwide glaucoma is the second most frequent eye disease and the leading cause of irreversible blindness. There are two major types, primary open angle (POAG) in which the iridocorneal angle is open, and angle closure glaucoma. POAG is usually presented as the most common type which accounts for three quarters of all glaucomas (see e.g., Kapetanakis et al., 2015).

GON can occur at any level of intraocular pressure (IOP) and, contrary to what had been thought previously, normal tension glaucoma seems to be quite frequent, may even comprise about one half of the POAG patients (Heijl, 2011). Moreover, among patients displaying increased IOP (>21 mmHg, the condition called ocular hypertension) the majority neither have, nor will ever develop symptomatic glaucoma. For example, in an Italian study which comprised 4927 subjects over 40 years of age, the overall prevalence of ocular hypertension, hypertensive POAG and normal tension glaucoma was 2.1%, 1.4% and 0.6%, respectively (Bonomi et al., 2001). In a Japanese study (Kitazawa et al., 1977) 75 patients with IOP above 21 mmHg were followed for over 9 years without treatment, and only seven of them (9.3%) developed glaucoma with visual field loss. Probably the longest follow-up of untreated ocular hypertension was reported in a Danish study, in which 41 persons with ocular hypertension and 38 persons with normal IOP were followed. Over 20 years the rate of glaucoma development reached 34% in initially hypertensive and 5% in initially normotensive individuals (Lundberg et al., 1987). Although elevated IOP appears to be the most important modifiable risk factor of glaucoma, certainly other factors are involved.

POAG is an indolent disease, developing over many years, with age being very important but unmodifiable risk factor. Its natural history can be presented as the “glaucoma continuum” which begins with acceleration of retinal ganglion cells (RGC) death, then progresses to detectable change in the retinal nerve fiber layer (RNFL) and narrowing of the visual field, and ultimately leads to blindness (Weinreb et al., 2004). In a recent article (Wollstein et al., 2012) RNFL thickness at which visual field damage becomes detectable has been determined, and it was concluded that a substantial structural loss (~17%) is necessary to cause functional loss detectable with the current testing methods of standard automated perimetry. The rate of disease progression is very variable, although the opinion has been presented that the mean rates of visual field deterioration depend on disease subtype, being the slowest in normal tension glaucoma and the fastest in exfoliation glaucoma (Heijl, 2011).

During the major part of the glaucoma continuum the disease is either undetectable, or asymptomatic, which may be termed “preglaucoma”. It has been argued that screening for a predisease (i.e., a disease in the asymptomatic phase) would make sense only if the following three conditions are met: (1) patients designated as having it are far more likely to develop symptomatic disease than the rest of the general population; (2) there is a feasible intervention that effectively reduces the likelihood of developing full-blown disease; (3) benefits of intervening on predisease outweigh harms. Currently there is no screening test for pre-glaucoma which fulfills these requirements (Viera, 2011). Screening is also not recommended because of potential harm of overdiagnosis and overtreatment of people who would never develop noticeable vision problems (Moyer et al., 2013). A technique which might provide a sensitive biomarker for retinal ganglion cell function and could be used to identify patients in whom glaucoma damage is incipient before visual field changes occur, is pattern electroretinography (PERG), a test that measures the electrical activity generated by retinal cells in response to a standardized light stimulus (Bach and Hoffmann, 2008). Unfortunately PERG is technically demanding and not suitable for routine ophthalmic diagnostics.

Pharmacological treatment of POAG, which should be reserved to symptomatic patients, is presently based exclusively on topically applied drugs that decrease IOP by decreasing aqueous production, increasing its outflow, or both. The most popular medications are prostaglandin analogs (e.g., latanoprost), β-blockers (e.g., timolol), a carbonic anhydrase inhibitor (dorzolamid) and a selective α2 adrenergic receptor agonist (brimonidine). However, treatment of POAG is frequently ineffective. Randomized clinical trials have demonstrated progression of the disease despite significant IOP lowering; older age and greater IOP fluctuation increased the odds of visual field narrowing progression (Nouri-Mahdavi et al., 2004). In a real life the major problem is poor adherence, its rates reported as 30–80%. In a recent article (Newman-Casey et al., 2015) among 11 major reasons of nonadherence to glaucoma therapy the following were identified as the most important: decreased self-efficacy, forgetfulness and difficulty with instilling drops.

An important aspect of glaucoma is that the disease is not confined to the RGC layer of retina and to the optic nerve, but it spreads along the entire retino-geniculo-cortical visual pathway, up to the visual cortex (Yücel and Gupta, 2008; Bogorodzki et al., 2014). Whereas it is generally believed that the disease starts at the level of retina from where the degeneration expands along the optic nerve, the index of global visual field damage obtained with a Humphrey perimeter (called the mean deviation, MD, and expressed in dB) may not be exclusively related to a retinal dysfunction, but may also reflect an impairment at the supraretinal level, detectable as an additional prolongation of visual evoked potentials (VEP; Parisi, 2001). It has been postulated that treatment of glaucoma should not be confined to the RGC but should also be directed toward glaucomatous degeneration-susceptible neurons in the brain (Gupta and Yücel, 2007). Of note in this context is that the recent neuroimaging data suggest that cerebral microinfarcts may be an intracerebral risk factor for glaucomatous optic nerve atrophy (Schoemann et al., 2014).

## Mechanisms of RGC Death and Biomarkers of Glaucoma

The hallmark of glaucoma is the selective death of RGC, neurons which “give birth” to axons comprising the optic nerve. Retina is a part of the central nervous system, and some authors suggested that, at the molecular level, glaucoma is in many respects similar to chronic central neurodegenerations like e.g., Alzheimer’s disease (see e.g., Jindal, 2015). Experimental studies on retinal cultures and animal models such as chronically increased IOP, RGC axotomy or partial optic nerve crush show that selective RGC death evoked by elevation of IOP or injury to the optic nerve may occur for several reasons such as excitotoxicity, oxidative stress, failure of axonal transport and neurotrophic factor deprivation, mitochondrial dysfunction, etc. (Almasieh et al., 2012; Osborne and del Olmo-Aguado, 2013). A popular model to study mechanisms of glaucomatous RGC loss is DBA/2J mice which spontaneously develop iris atrophy with pigment dispersion, and glaucoma (John et al., 1998). Many non-clinical results are corroborated by data on biochemical biomarkers in glaucoma patients (see, for example Pinazo-Durán et al., 2013). Some mechanisms currently considered of major importance in glaucomatous RGC death are outlined below.

### Excitotoxicity

Glutamate, a leading excitatory neurotransmitter in the brain and retina, may act as a neurotoxin. In fact, the first observation of glutamate neurotoxicity concerned toxicity of sodium glutamate given peripherally toward cells of the inner layers of retina (Lucas and Newhouse, 1957). The concept of excitotoxicity as a pivotal pathomechanism of various acute and chronic neurodegenerative diseases has been worked out subsequently.

Several *in vitro* and *in vivo* studies have shown that retinal exposure to glutamate or its analog N-methyl-D-aspartate (NMDA) is toxic to RGC (reviewed in Barkana and Belkin, 2004). Although in some experiments acute intraocular NMDA application produced only transient and reversible loss of vision (Sabel et al., 1995), more relevant to human POAG seemed to be chronic low-dose elevation in vitreal glutamate, which was toxic to ganglion cells when maintained for 3 months (Vorwerk et al., 1996). Purified RGC* in vitro* were found resistant to NMDA or glutamate-induced cell death (Ullian et al., 2004). Yet two other subtypes of glutamate ionotropic receptors, AMPA and kainate receptors, are present inside RGC synapses (Sethuramanujam and Slaughter, 2015) and may participate in RGC death, in cooperation with inflammatory mediators such as matrix metalloproteinase-9 (Zhang et al., 2004) and tumor necrosis factor alpha (Cueva Vargas et al., 2015). The finding of a twofold elevated glutamate level in the vitreous of glaucoma patients and also monkeys in which glaucoma had been experimentally induced (Dreyer et al., 1996) provided further argument to support the hypothesis that excitotoxicity is involved in this disease.

### Oxidative and Nitrosative Stress

These terms describe a situation in which tissue generation of oxidizing free radicals (reactive forms of oxygen and nitrogen) exceeds the means of inactivating these, which results in accumulation of dysfunctional, oxidatively damaged proteins, lipids and nucleic acids. A wealth of data supports the hypothesis of an important role of oxidative stress in various preclinical models of glaucoma (reviewed by Tezel, 2006).

In retina and optic nerve oxidative/nitrosative stress may be evoked by glutamate excitotoxicity, or may occur for other reasons, such as dysfunction of mitochondria. Signs of decreased antioxidant defenses and increased levels of pro-oxidants have also been identified in the aqueous humor, ocular tissues and blood of glaucoma patients. For example, Sorkhabi et al. (2011) reported significantly increased levels of a marker of DNA oxidative damage 8-hydroxy-deoxyguanosine and decreased total antioxidant status in both aqueous humor and serum of POAG patients compared to those with cataracts. Gherghel et al. (2013) reported on low systemic glutathione levels in blood of patients suffering from primary open angle hypertensive as well, as normotensive glaucoma, indicating a compromise of the antioxidant defense systems.

### Failure of Axonal Transport and Neurotrophin Deprivation

RGC axons traverse a large distance; anterograde continuous delivery of organellae such as mitochondria and also proteins from RGC somas is required for maintaining their function. Impairment of both the anterograde and retrograde axonal transport is considered an important pathogenic feature of glaucoma (Fahy et al., 2016).

In particular, brain-derived neurotrophic factor (BDNF), along with neurotrophins −4 and −5 (NT-4/5) is vital for maintaining RGCs in the retina. Its trophic effects are primarily mediated through high-affinity plasma membrane receptor TrkB. BDNF and TrkB are widely expressed in the RGCs and lamina cribrosa. Activation of TrkB activates several pro-survival kinases, including Akt and extracellular signal-regulated kinases 1 and 2 (Erk1/2). BDNF is produced locally by RGCs and astrocytes in the retina, but it is also synthesized in the superior colliculus and the lateral geniculate nucleus, from where it is retrogradely transported through RGC axons to the retinal ganglion cell bodies (Harvey et al., 2012).

In two small studies BDNF levels were found significantly lower in sera (Ghaffariyeh et al., 2011) and tears (Ghaffariyeh et al., 2009) of patients with POAG, compared to age-matched controls without any apparent ocular or systemic diseases. Although lower BDNF level in glaucoma could be related to extraocular reasons such as, for example, more sedentary lifestyle of glaucoma patients (exercise may increase serum and plasma BDNF level (Coelho et al., 2013), these data correspond to the concept of BDNF insufficiency in glaucoma.

### Mitochondrial Dysfunction and Apoptosis

Proper function of the retina and optic nerve is critically dependent on mitochondria that supply ATP necessary to support their function and structure. Primary and secondary impairments of these organelles have been shown in cell and animal glaucoma models and in human glaucoma (Lee et al., 2011).

In experimental models of glaucoma abundant evidence has been provided that RGC die through apoptosis (Garcia-Valenzuela et al., 1995; Schuettauf et al., 2004). Apoptosizing RGC were found in human POAG (Kerrigan et al., 1997), and also in anterior ischemic optic neuropathy (Levin and Louhab, 1996). Apoptosis as the primary pathway of RGC death in glaucoma and involvement of mitochondria-generated free radicals in this pathology are currently generally accepted, although details such as involvement of caspases (Tezel and Yang, 2004) and possible contribution of other mechanisms such as autophagy (Wang et al., 2015) are debated.

In summary it may be concluded that, in response to glaucomatous stimuli, cross talk between proapoptotic signals (caspase activation and mitochondrial dysfunction) and survival promoters (neurotrophins) determines the fate of RGCs (Pinazo-Durán et al., 2013).

## Citicoline: A Neuroprotectant Acting Through Mechanisms Relevant to Glaucoma

The concept of neuroprotection is that structure and function of diseased neurons can be rescued by interfering with mechanisms of injury and death pathways. Already 20 years ago Schwartz et al. (1996) pointed out to the accumulating evidence suggestive of that, regardless of the primary trigger of the retinal and optic nerve damage in glaucoma, the disease will continue to progress even when the cause is removed; therefore substances which are neuroprotective may be useful for the treatment of this disease. Since then the idea of complementing IOP lowering in POAG by neuroprotective treatment has been endorsed by many authors (two recent examples: Chang and Goldberg, 2012; Song et al., 2015). Neuroprotective agents should be helpful in preserving visual function through correcting the imbalance between cellular death and survival signals and preventing RGC death and optic nerve damage (Sena and Lindsley, 2013).

Neuroprotective properties of citicoline have been shown in various experimental paradigms. Some of them were models of glaucoma, the others modeled brain neurodegenerative diseases in which citicoline seemed to counteract various pathological mechanisms believed to be involved also in glaucomatous RGC loss.

### Rescue of RGC in Partial Optic Nerve Crush

Partial crush injury of the rat optic nerve has been developed as a model of progressive degeneration of RGCs which recapitulates much slower degeneration that occurs in POAG (Schwartz, 2005). After the short initial phase of immediate death following mechanical axotomy of some ganglion cells, the situation in the retina and the optic nerve attains characteristics similar to a chronic disease. Both in glaucoma and in the partial optic nerve crush model the selective and delayed RGC death occurs, whereas the other cellular layers of the retina are spared. Many ganglion neurons, although their axons are not damaged by an acute insult, eventually degenerate as a consequence of the degenerative environment produced by the initial injury. While it is rather certain that a primary insult initiating neurodegeneration of RGCs in glaucoma is different from that which initiates RGCs death after the callibrated partial optic nerve crush, it has been assumed that in both cases the self-perpetuating secondary degeneration of RGCs results from a similar hostile environment generated by dying neural cells, which consists of mediators of oxidative stress, free radicals, excessive extracellular glutamate and calcium ions, and other factors. Therefore partial optic nerve crush is a quick and relatively simple experimental paradigm frequently used to assess neuroprotective efficacy of potential anti-glaucoma drugs (Li et al., 2014).

Given after partial optic nerve crush, citicoline was found effective in rescuing RGC connected to their brain targets (Schuettauf et al., 2006). Similar effects were obtained with lithium (Schuettauf et al., 2006), which has recently been identified as a potential neuroprotective agent in central neurodegeneration (Lazzara and Kim, 2015). Neuroprotection provided by citicoline and lithium were not additive, moreover both compounds similarly increased retinal expression of the apoptotic regulating protein Bcl-2, indicating one of the mechanisms which may be engaged in the neuroprotective effect of both citicoline and lithium (Schuettauf et al., 2006). A possible explanation may be that lithium upregulates BDNF (see Lazzara and Kim, 2015), whereas citicoline may act as a BDNF mimic (see below).

### Counteracting Excitotoxicity

Although *in vitro* exposure of cultured RGCs to glutamate or NMDA, or intravitreal injection of these excitotoxins were frequently employed for testing neuroprotective efficacy of various compounds, citicoline has not been tested with any of these experimental paradigms. However, neuroprotective effects of citicoline in the adult rat retina were described after intravitreal injection of kainic acid (KA). Han et al. (2005) evaluated effects of citicoline on KA-induced changes in morphometric features of cells and expression of neuronal and endothelial isoforms of nitric oxide synthase (NOS). In the second article thickness of various retinal layers were measured and immunochemical methods were used to visualize the expression of choline acetyltransferase and tyrosine hydroxylase (Park et al., 2005). Both types of analysis revealed significant attenuation of KA-induced damage in retinas from citicoline-treated animals. An important finding was that intravitreal KA resulted in increased expression of NOS isoforms, and citicoline decreased this effect, apparently counteracting nitrosative stress. In the third report (Park et al., 2006) citicoline was found to decrease ERK1/2 kinase activation caused by KA.

### Anti-Apoptotic Effects and Mimicking Neurotrophic Factors

Using murine retinal explants Oshitari et al. (2002) have shown that citicoline can rescue damaged RGCs through an anti-apoptotic effect and also can support neurite regeneration of damaged RGCs. In the later report from the same laboratory (Oshitari et al., 2010) effects of neurotrophic factors (BDNF, NT-4) or citicoline on neuronal apoptosis and neurite regeneration in cultured rat retinas exposed to high glucose (HG) are described. The study showed that BDNF, NT-4, and to some extent also citicoline reduced the retinal neuronal apoptosis induced by HG and increased the number of regenerating neurites. These effects were correlated with the reduction of the expression of active forms of caspases-9 and -3. (It is worth to mention that citicoline prevented brain neuronal caspase-3 activation also in other *in vivo* models of neurodegenerative diseases such as rat models of perinatal brain asphyxia (Fiedorowicz et al., 2008) and adult brain ischemia (Krupinski et al., 2002; Takasaki et al., 2011)). Recently it has been confirmed that in rat primary retinal cultures exposed to toxic levels of glutamate or glucose citicoline counteracts neuronal cell damage by decreasing proapoptotic effects and counteracting synapse loss (Matteucci et al., 2014).

### Retarding Senescence of Brain Mitochondria

During aging various impairments in mitochondria from different rat brain areas occur. Intraperitoneal treatment of aged rats with citicoline at a dose of 20 mg/kg body weight per day for 1 month caused reversal of some of these age-dependent changes (Villa et al., 1993). These effects were attributed to increased availability of cytidilic nucleotides required for the synthesis of membrane phospholipids phosphatidylserine and phosphatidylethanolamine and/or to the improvement of brain energy metabolism. Interestingly, the treatment was effective in 18 month old, but not in 24 month old animals (Villa et al., 2012), suggesting that citicoline can retard but will not reverse advanced mitochondrial aging.

Support for the hypothetical positive influence of citicoline on aging brain mitochondria comes also from a human study in which 31-phosphorous magnetic resonance spectroscopy has been used to characterize the effects of this compound on high-energy phosphate metabolites and constituents of membrane synthesis in the cortical frontal lobe of middle aged human volunteers. Significant increases in phosphocreatine, ATP, the ratio of phosphocreatine to inorganic phosphate, and changes in membrane phospholipids were observed after 6 weeks of citicoline treatment, suggestive of that citicoline improves bioenergetics and phospholipid membrane turnover in a brain region critical for cognition and memory. Citicoline supplementation may therefore help to mitigate cognitive declines associated with aging by increasing energy reserves and utilization, as well as increasing the amount of essential phospholipid membrane components needed to synthesize and maintain cell membranes (Silveri et al., 2008). We may take it for granted that mitochondria are involved in these improvements.

### Effects on Non-Glutamatergic Neurotransmitter Systems

In the previous review on citicoline and glaucoma (Grieb and Rejdak, 2002) we indicated data showing that, along with neuroprotective properties, citicoline injections increased the levels and enhanced the rate of synthesis of acetylcholine, dopamine, noradrenaline and serotonin in some brain areas. To find out whether similar increases occur in retina, we treated rabbits with 50 mg/kg citicoline intraperitoneally for a week; following this treatment, significantly increased retinal dopamine were noted. We concluded that, considering its key role in visual information processing, elevation of the retinal dopamine may be responsible, at least partially, for the improvement of visual function following citicoline treatment of patients with glaucoma (Rejdak et al., 2002).

It shall be also mentioned that citicoline is an intracellular donor of choline which mimics electrophysiological and pharmacological effects of acetylcholine at the alpha7 nicotinic acetylcholine receptors (α7 nAChR; Alkondon et al., 1997). For this reason combining citicoline with galantamine, a positive allosteric modulator of these receptors, has been proposed as a new approach to schizophrenia treatment (Deutsch et al., 2013). Recently a new synthetic α7 nAChR agonist has been shown to prevent loss of RGC in a rat model of glaucoma evoked by increased IOP (Iwamoto et al., 2014). It is, therefore, possible that one of the mechanisms by which citicoline rescues RGC in glaucoma may involve α7 nAChR.

### Effects on Demyelination and Remyelination

Recently beneficial effects of citicoline have been demonstrated in experimental murine models of autoimmune encephalomyelitis (EAE) and cuprizone-induced demyelination (Skripuletz et al., 2015). Qualitatively similar effects of this compound had been reported previously in rat EAE models, although only in the abstract form (see Grieb, 2015b).

EAE is a model of multiple sclerosis (MS), which is a prototypical demyelinating disease. Of note is that patients suffering from active MS, particularly early in the disease course, exhibit accelerated thinning of the ganglion cell/inner plexiform layer in the retina (assessed by the optical coherence tomography; Ratchford et al., 2013). These findings are suggestive of retinal changes in MS reflecting global CNS processes. Effects of citicoline in MS patients have not been evaluated yet, but such trial is certainly indicated. Counteracting demyelination and promoting remyelination may also be relevant in glaucoma; some clinical neuroimaging data on POAG patients brains present signs of demyelination (Michelson et al., 2013) which may precede ultimate axonal degeneration.

## Citicoline for Glaucoma: Clinical Evidence

**Figure 1:**
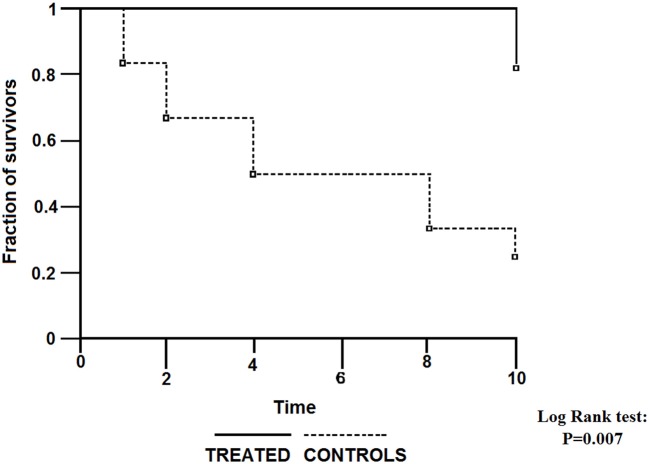
**Survival curves for visual field worsening of primary open angle glaucoma (POAG) patients treated with intraocular pressure (IOP)-decreasing topical therapy with or without addition of citicoline given intramuscularly.** (Reproduced with permission from John Wiley & Sons, Inc., Virno et al., 2000).

The first report on treatment of POAG patients with intramuscular citicoline injections was published more than a quarter of century ago (Pecori Giraldi et al., 1989). The follow-up of this initial study (Virno et al., 2000) showed an excellent outcome of the therapy continued for 10 years: only 2 of 11 patients treated with repeated courses of intramuscular citicoline injections (1 gram daily for subsequent 15 days, repeated every 6 months), but 5 of 12 patients who did not receive citicoline injections encountered decreased fraction of the visual field by at least 500 mm^2^, as assessed by the Video Screen Perimetry (Figure 1).

Ten years after the aforementioned first report, results of a double-blind, placebo-controlled study were published in which electrophysiological methods (PERG and VEP) were used to assess function of retinal and supraretinal parts of the visual pathway. This study showed significant in both electrophysiological indices of the function of visual pathway following a course of intramuscular injections of citicoline (Parisi et al., 1999). Since the improvements were transient, treatment courses had to be repeated every few months. Long-term beneficial effects of repeated courses of citicoline injections in POAG patients were later confirmed by the electrophysiological study with follow-up of 8 years (Parisi, 2005).

**Figure 2:**
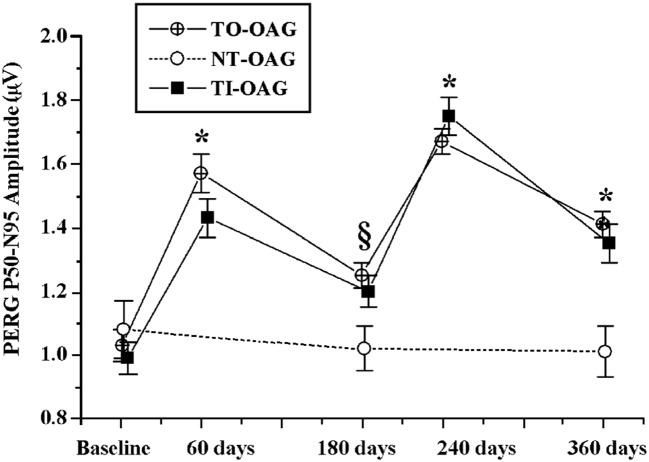
**Comparison of changes in amplitudes of pattern electroretinograms in POAG patients treated with a topical IOP-decreasing therapy non-treated open angle glaucoma (NT-OAG), and treated with citicoline intramuscularly open angle glaucoma (TI-OAG) or orally open angle glaucoma (TO-OAG).** *p < 0.01, ^§^*p* > 0.01. (Reproduced with permission from Elsevier, Inc., Parisi et al., 2008).

Because injections are not a preferred treatment for ophthalmic patients suffering from a chronic disease, in a small pilot study we tested effects of two biweekly courses of citicoline given orally in a dose of 1 gram per day, separated by a 2 week break, on VEPs in POAG patients (Rejdak et al., 2003). Although the study group was small (21 eyes in 11 patients), highly significant shortening of VEP100 latency, and less marked but also significant increase in VEP amplitude were found. Later the effects of citicoline given by intramuscular injections (1 gram daily) or orally (1.6 gram daily) were compared in the larger Italian study (Parisi et al., 2008), and the two treatment modalities were found equivalent. Two months of intramuscular or oral citicoline doses repeated daily resulted in improvements in both PERG and VEP; although the positive effects almost vanished after the next 4 months during which no citicoline was taken, resumption of the treatment produced even somewhat larger improvements (Figure 2). The authors concluded that treatment with citicoline is, indeed, neuroprotective, as it seems to halt glaucoma progression, but is not curative and must be periodically repeated to uphold beneficial effects.

**Figure 3:**
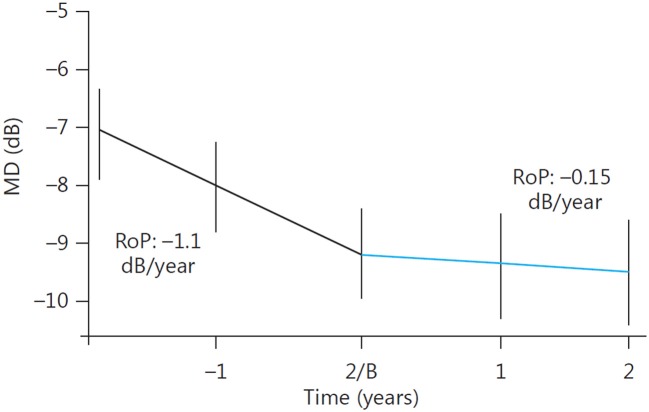
**Change in the rate of progression (RoP) of POAG following addition of oral citicoline to a topical antiglaucoma therapy.** B, Baseline. (Reproduced with permission from Karger Publishers, Ottobelli et al., 2013).

Yet another confirmation of long-term beneficial effects of oral citicoline was provided by the study performed in three Italian university clinics, in which 41 patients diagnosed as having progressing POAG despite “controlled IOP” were treated with oral citicoline solution, dose 500 mg per day for 4 months, divided by 2-month no-treatment periods (Ottobelli et al., 2013). The study was not randomized, but its prospective part was preceded with retrospective assessment of the pre-treatment rate of progression of the disease as measured with a Humphrey perimetry. The rate of progression before treatment initiation was −1.1 dB/year, and during citicoline treatment it significantly decreased to −0.15 dB/year (Figure 3).

Recently Parisi et al. (2015) reported on the treatment of POAG patients with topical application of citicoline in the form of eye drops. Although the results seemed to be similar to that of oral citicoline, the idea of topical delivery of citicoline may not be endorsed. Citicoline is water soluble and will poorly penetrate cornea. The authors argue that preclinical data show that citicoline reaches the vitreous provided that high molecular weight hyaluronic acid and benzalkonium chloride (BAC) are used as penetration enhancers. BAC, which is currently the most popular preservative for eye drops, is blamed for inducing ocular surface changes and several other side effects (Baudouin et al., 2010). Oral citicoline, usually devoid of any side effects, would be much more acceptable.

Parenthetically, it is worth to mention that citicoline displays nootropic properties and its intake is beneficial to patients with subjective memory disorders or mild or moderate vascular cognitive impairment (Fioravanti and Yanagi, 2005). We may thus expect that taking citicoline concomitantly with IOP-decreasing eye drops might effect in improved adherence to glaucoma therapy.

## Citicoline for Glaucoma in Light of the Evidence-Based Medicine

In an article discussing analogies between senile dementia and glaucoma, Jain and Aref (2014) suggested that in both diseases neuroenhancement may be sought by using agents such as citicoline, currently available as an over the counter supplement. On the other hand the EFSA Panel on Dietetic Products, Nutrition and Allergies, delivering an opinion on the scientific substantiation of a health claim related to the new food cytidine 5-diphosphocholine and maintenance of normal vision in elderly subjects since middle age concluded that a cause and effect relationship has not been established between the consumption of CDP-choline and maintenance of normal vision, therefore the aforementioned health claim cannot be supported (EFSA Panel on Dietetic Products Nutrition and Allergies NDA, (2014)). We may describe these two contrasting attitudes as permissive and restrictive, respectively. Somewhat paradoxically, in the aforementioned examples permissive attitude concerns the use of citicoline in symptomatic disease (glaucoma), whereas restrictive attitude concerns its intake by asymptomatic general population for possible prophylaxis of this disease.

Implicit arguments for the restrictive attitude towards oral citicoline for maintenance of healthy vision were probably similar to those referred to by the evidence-based medicine (EBM) in the case of drugs. As the name suggests, EBM is a code of conduct based on using evidence to make clinical decisions. A cornerstone of EBM is the hierarchical system of classifying evidence, known as the “levels of evidence pyramid”, which is directly translatable to the “grades of recommendation” (Shekelle et al., 1999). The “gold standard” of high-grade evidence is result of prospective, randomized and blinded, so-called phase III drug trials with properly designed clinical end points, their metaanalyses and systematic reviews. Evidence based on non-randomized clinical studies is considered much less convincing, and recommendations based on various non-clinical studies (frequently called, somewhat contemptuously, “bench research”) are not trusted at all.

Undoubtedly, restrictive attitude is praiseworthy toward drugs which are considerably toxic and very expensive (these are typical attributes of the so-called “originator drugs”). Indeed, to obtain market authorization for a new drug, currently it is necessary to demonstrate with a randomized and blinded study that potential risks related to treatment are outweighed by therapeutic efficacy. However, in the case of a food supplement such requirements are undue. If not excessively ingested, food supplement is considered totally risk-free, therefore the issue of efficacy outweighing risks is irrelevant. In the case of citicoline, the maximum daily dose established by EFSA as totally safe is 500–1000 mg.

Also, it is unrealistic to expect that a cause and effect relationship will be established between intake of a food supplement and its prophylactic or therapeutic efficacy. For example, although yearly world production of vitamin C is estimated as 110,000 tons (Pollak, 2011), its efficacy in preventing and treating the common cold is a subject of controversy for more than 70 years and the issue is still not solved (Hemilä and Chalker, 2013). In the case of POAG, obtaining conclusive clinical data on the efficacy of prophylaxis with a dietary supplement would be even further impeded by lack of a screening test and the indolent time course of the disease.

In the *Glaucoma Book*, a comprehensive review of knowledge on glaucoma published in 2010 by Springer, citicoline is briefly mentioned only once, in a chapter written by Zelefsky and Ritch (2010), devoted to the so-called “alternative” and “non-traditional” substances usually available over the counter, such as alpha-lipoic acid, fish oil and omega-3 fatty acids, carnitine and the like. In the introduction to their chapter the authors state that in the absence of clinical trials concerning these natural compounds, it devolves upon the readers to make the best possible guess as to what might or might not be effective in glaucoma. As we tried to show, the wealth of data on neuroprotective properties of citicoline, including some clinical data, is quite large and convincing. This relatively old nootropic drug which recently turned out to be a food supplement seems to be a valuable addition to the conventional treatment, and also a rational option for prophylaxis of POAG.

## Author Contributions

PG originated the concept of the review, and was responsible for its design and contents relating to non-clinical aspects. AJ and MR were responsible for presentation of clinical aspects of glaucoma pathophysiology and treatment. RR was responsible for integrating non-clinical and clinical aspects of the reviewed subjects.

## Conflict of Interest Statement

The authors declare that the research was conducted in the absence of any commercial or financial relationships that could be construed as a potential conflict of interest.
